# SnRK1 and TOR: central regulators of autophagy in plant energy stress responses

**DOI:** 10.1007/s42994-025-00218-3

**Published:** 2025-05-15

**Authors:** Lei Feng, Xibao Li, Xuan-Ang Zheng, Zhao Zheng, Qing-Ren Liu, Chuanliang Liu, Qian-Lin Zhu, Wenjin Shen, Chao Yang, Hongbo Li, Xiaorong Wan, Yixiong Zheng, Jun Zhou, Caiji Gao

**Affiliations:** 1 Guangdong Provincial Key Laboratory of Biotechnology for Plant Development, School of Life Sciences, MOE Key Laboratory & Guangdong Provincial Key Laboratory of Laser Life Science, and College of Biophotonics, South China Normal University, Guangzhou, 510631 China; 2 Guangdong Provincial Key Laboratory of Applied Botany, South China Botanical Garden, Chinese Academy of Sciences, Guangzhou, 510650 China; 3 Guangzhou Key Laboratory for Research and Development of Crop Germplasm Resources, Zhongkai University of Agriculture and Engineering, Guangzhou, 510225 China

**Keywords:** *Arabidopsis thaliana*, Autophagy, Energy stress, FLZ, SnRK1, TOR

## Abstract

Plants harness light through photosynthesis to produce chemical energy, a cornerstone of life on Earth. However, environmental challenges, such as insufficient light, nutrient deficiencies, and abiotic stresses, often disrupt energy availability, compelling plants to activate autophagy. This process degrades superfluous or damaged cellular components to recycle building blocks for vital functions. Like animals and yeast, plants employ conserved energy-sensing pathways, notably the antagonistic SNF1-related kinase 1 (SnRK1; homologous to AMP-activated protein kinase in animals) and target of rapamycin (TOR) signaling cascades. Plants have also evolved unique strategies to initiate autophagy when faced with energy stress. Recent studies have elucidated plant-specific mechanisms, including the pivotal role of FCS-like zinc finger proteins in integrating stress and metabolic signals to modulate SnRK1 and TOR activity. This review synthesizes the current understanding of autophagy regulation in plants under energy stress, emphasizing how SnRK1 and TOR orchestrate cellular homeostasis. It also examines organelle-phagy—chlorophagy, mitophagy, and lipophagy—in sustaining energy balance during stress. Amid intensifying climate challenges, including drought, nutrient scarcity, and erratic weather, elucidating these mechanisms is critical for engineering crops with enhanced resilience and productivity, thereby addressing global food security challenges. Furthermore, as autophagy is conserved across eukaryotes, plant research offers insight into universal cellular processes, potentially informing applications related to human health. This review also consolidates recent advances and proposes future research to deepen our understanding of energy signaling and autophagy in plants.

## Introduction

Energy stress in plants occurs when the demand for energy exceeds the available resources, often due to environmental factors, such as nutrient deficiency, extreme temperatures, drought, or other conditions that disrupt energy generation or utilization. This stress affects key cellular pathways, including photosynthesis, respiration, and growth, ultimately compromising plant development and productivity. In response, plants adjust their physiology, metabolism, and resource allocation, while activating stress-response mechanisms (Feng et al. 2025; Li et al. 2024b). To sense and respond to energy stress, plants have evolved complex signaling networks involving phytohormones, transcription factors, and kinases to regulate metabolism and activate the expression of stress-responsive genes (Baena-González et al. 2007). A key response to energy stress is autophagy, a widespread cellular recycling mechanism responsible for the degradation of damaged or dysfunctional organelles and proteins, which releases energy and supports plant cell survival and function (Feng et al. 2024; Li et al. 2022b, 2024b).

Plants cope with energy stress by activating mechanisms that ensure their survival. Key energy sensors, including the kinases SNF1-related kinase 1 (SnRK1) and target of rapamycin (TOR), regulate various aspects of plant metabolism, thereby activating energy-conserving strategies and stress responses (González et al. 2020). The functions of SnRK1 and TOR are evolutionarily conserved across species. TOR is highly conserved in plants, especially in terms of its amino acid sequence. However, differences in rapamycin sensitivity between algae and land plants may stem from variations in the sequences of homologs for the TOR-interacting protein 12-kDa FK506-binding protein (FKBP12) or TOR itself, although experimental evidence is lacking (Díaz-Troya et al. 2011; Xiong and Sheen 2012). Polyploid species often contain multiple copies of *TOR*: The genomes of soybean (*Glycine max*), black cottonwood (*Populus trichocarpa*), and rapeseed (*Brassica rapa*) each contain two copies, while that of allotetraploid cotton (*Gossypium hirsutum*) has four (Song et al. 2017). Similarly, SnRK1 is conserved among plants, with bryophytes such as *Physcomitrium patens* having expanded families encoding the *α* subunit due to whole-genome duplication events, leading to expanded gene families. Land plants, particularly polyploids, often possess multiple SnRK1 *α* subunit genes, with some species encoding truncated isoforms lacking the ubiquitin-associated (UBA) domain or regulatory C-terminal domain (α-CTD), potentially conferring specialized functions (Jamsheer et al. 2019).

The energy status of plant cells is primarily reflected by the ratios of adenosine monophosphate/adenosine triphosphate (AMP/ATP) and ADP/ATP, which serve as critical indicators of cellular energy availability (Tyutereva et al. 2022; Xiao et al. 2024). When energy levels are low, the concentration of AMP increases relative to that of ATP, signaling an energy deficit. Similarly, the ADP/ATP ratio rises when ATP is consumed, further indicating a depletion of cellular energy reserves (Tyutereva et al. 2022). These ratios are sensed by SnRK1, which is activated under low-energy conditions, such as starvation or hypoxia, to inhibit energy-consuming biosynthetic pathways and upregulate catabolism, including autophagy, thereby releasing nutrients and metabolites to restore energy balance (Baena-González et al. 2007; Cho et al. 2019; Crepin and Rolland 2019; Im et al. 2014; Ramon et al. 2019). By contrast, TOR is activated when energy and nutrients are abundant, promoting anabolic programs such as protein synthesis and cell growth, while suppressing autophagy to ensure efficient energy utilization (Dong et al. 2022; Shi et al. 2018). Autophagy plays a critical role by recycling cellular components to generate energy upon energy stress, while alternative metabolic pathways such as fermentation and the use of stored reserves help maintain energy balance. Additionally, plants adjust photosynthetic efficiency, use reactive oxygen species (ROS) for signaling, and regulate stomatal opening to optimize water use and energy consumption (Yamauchi et al. 2019). These acclimation responses allow plants to maintain growth and function even under challenging environmental conditions. The integration of stress-responsive signaling pathways through TOR and SnRK1 helps coordinate autophagy and metabolic adjustments to energy status, allowing plants to dynamically respond to ever-changing environmental cues (González et al. 2020; Han et al. 2024; Pu et al. 2017; Soto-Burgos and Bassham 2017; Yang et al. 2019).

FCS-like zinc finger (FLZ) proteins, a unique class of C2-C2-type zinc finger proteins found exclusively in land plants, link the TOR and SnRK1 pathways (Chen et al. 2021; Jamsheer et al. 2015; Wang et al. 2023a). FLZs physically interact with the regulatory subunits of the SnRK1 complex, serving as adaptors that modulate the activity and localization of this energy-sensing kinase (Jamsheer et al. 2018b). When energy levels are low and SnRK1 is activated, FLZs may help recruit SnRK1 to specific cellular compartments and substrates, allowing it to phosphorylate key regulatory proteins that induce autophagy (Yang et al. 2023b). FLZs can also interact with components of the TOR kinase complex (Artins and Fernie 2023). The binding of FLZs to TOR subunits is thought to help fine-tune the balance between the antagonistic SnRK1 and TOR signaling pathways, ensuring a dynamic and coordinated response to plant energy status. By bridging the SnRK1 and TOR complexes, FLZs integrate diverse stress and metabolic signals (Artins and Fernie 2023), allowing plants to rapidly adjust their physiology and resource allocation in response to shifting environmental conditions through the precise modulation of autophagy and other metabolic pathways. The land plant-specific nature of FLZs underscores their importance in the evolutionary adaptation of plants to the terrestrial environment.

In this review, we discuss the intricate interplay involving TOR and SnRK1 in response to energy stress, with a focus on the distinct roles of plant-specific FLZs as potential energy sensors in modulating TOR and SnRK1 activities. We also discuss the roles of mitophagy, chlorophagy, and lipophagy in plants under energy limitation. Our goal is to provide a deeper understanding of the physiological and molecular regulatory networks in plants under energy stress conditions.

## TOR negatively regulates autophagy in plants

TOR functions as a sensor and positively regulates many cellular programs. Rapamycin, an antibiotic produced by the aerobic Gram-positive soil bacterium *Streptomyces hygroscopicus*, was discovered on Easter Island in the 1960s when the microbiologist Georges Nógrády was investigating why the islanders did not contract tetanus from walking barefoot (Powers 2022). Subsequently, rapamycin was shown to inhibit fungal growth and possibly also serve as an immunosuppressant and anticancer agent in humans (Loewith and Hall 2011). Genetic screening in the budding yeast *Saccharomyces cerevisiae* led to the identification of its target protein, which was subsequently named TOR (Heitman et al. 1991). The structure of TOR is highly conserved in eukaryotes, including yeast, animals, and plants, and typically includes multiple domains: N-terminal HEAT repeats, a focal adhesion target (FAT) domain, an FKBP/rapamycin-binding domain, a kinase domain, and a C-terminal FATC domain (Cai et al. 2022).

**Fig. 1:**
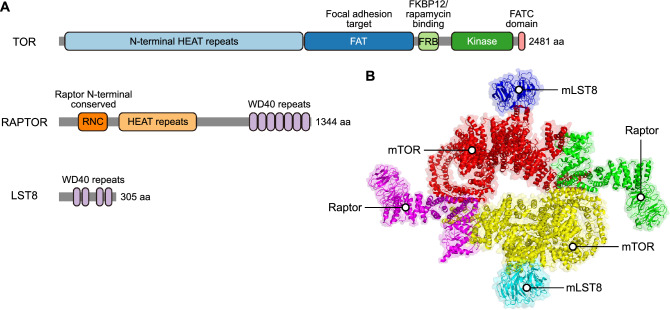
Structural features of TORC1 in plants and the architecture of mTORC1 in animals. **A** Structural features of the TORC1 subunits in *Arabidopsis*: TOR (UniProt accession number: Q9FR53), RAPTOR (UniProt accession number: Q93YQ1), and LST8 (UniProt accession number: Q9LV27). Functional domains were annotated using InterPro (https://www.ebi.ac.uk/interpro/) and manually verified based on the previous studies (Takahara et al. 2020; Yang et al. 2013). The gray rectangles represent the overall protein sequences, while colored rectangles or ellipses indicate the functional domains. **B** Structural model of the human mTORC1 complex, presented as both a cartoon representation and a transparent surface model. The model was derived from PDB:5H64 and manually annotated based on previous work (Aylett et al. 2016)

Functional studies revealed that TOR is a key positive regulator that coordinates cell proliferation, development, and metabolism in a spatiotemporal manner by integrating nutrient, energy, growth factor, and environmental signals (Meng et al. 2022). Notably, in plants, the deletion of TOR disrupts embryo development, leading to embryonic death, underscoring its critical role in developmental processes (Menand et al. 2002). In mammals, TOR functions in two distinct protein complexes: TOR complex 1 (TORC1) and TORC2 (Albert and Hall 2015). As summarized in Fig. 1, TORC1 is dimeric and consists of TOR, lethal with sec-13 protein 8 (LST8), and regulatory-associated protein of TOR (RAPTOR) (Albert and Hall 2015; Aylett et al. 2016). TORC1 is conserved between plants and mammals, with LST8 interacting with the kinase domain of TOR to bind to its substrates, while RAPTOR regulates the stability and kinase activity of the complex (Bertoni 2020; Salem et al. 2018). Mammalian TORC2 contains TOR, LST8, rapamycin-insensitive companion of TOR (RICTOR), and SAPK-interacting protein 1 (SIN1) (Saxton and Sabatini 2017). Plants lack homologs of RICTOR and SIN1, but this does not preclude the existence of other TOR complexes distinct from mammalian TORC2 (Pacheco et al. 2021; Tatebe and Shiozaki 2017).

TOR is activated under nutrient-sufficient conditions and inactivated under nutrient-limiting conditions (Wu et al. 2019). Glucose and glutamine, which provide carbon for the tricarboxylic acid cycle to produce ATP, positively regulate TORC1 activity (Kim et al. 2013). Under energy-sufficient conditions, the ATP-dependent RuvB-like AAA ATPase 1/2 (RUVBL1/2)–TEL2–TTI1–TTI2 (TTT) complex promotes obligate dimer formation and the lysosomal localization of TORC1 (Kim et al. 2013). Energy stress represses the RUVBL–TTT complex and promotes its degradation, thereby preventing its interaction with TOR (Kim et al. 2013). The RUVB–TTT complex is conserved between mammals and plants (Shi et al. 2018). AMP-activated protein kinase (AMPK; SnRK1 in plants) negatively regulates TOR activity by phosphorylating the TORC1 component RAPTOR (González et al. 2020; Nukarinen et al. 2016).

TOR inhibits autophagy in both yeast and mammals by preventing the formation of active autophagy initiation complexes under nutrient-rich conditions. In yeast, TORC1 phosphorylates Autophagy 13 (Atg13), a component of the Atg1 complex, which is essential for the initiation of autophagy. The phosphorylation of Atg13 by TORC1 disrupts its interaction with Atg1 (the yeast homolog of mammalian Uncoordinated-51-like kinase 1 [ULK1]), thereby blocking the formation of the active Atg1–Atg13–Atg17 complex. This inhibition impedes the recruitment of autophagy-related proteins to the phagophore assembly site (PAS), effectively suppressing autophagy (Ganley et al. 2009; Laplante and Sabatini 2012; Wong et al. 2013). Upon nutrient limitation or TORC1 inhibition in yeast, Atg13 is dephosphorylated, allowing it to bind to Atg1 and Atg17, thus activating autophagy (Wong et al. 2013). Most yeast genes involved in the TOR and autophagy pathways have homologs in humans and plants, suggesting a highly conserved relationship across eukaryotes (Díaz-Troya et al. 2008).

TOR also negatively regulates autophagy in plants. For instance, knockdown of *TOR* resulted in the upregulation of *ATG* genes and the constitutive activation of autophagy in Arabidopsis (*Arabidopsis thaliana*) (Liu and Bassham 2010). Whether TOR also directly inhibits ATG13 phosphorylation in plants remains to be investigated (Xiong and Sheen 2014). In yeast, TOR regulates the activity of the Rab GTPase Yeast protein two 1 (Ypt1, also reported as Rab1), an essential regulator of autophagy. TOR directly phosphorylates Ypt1, thereby preventing Ypt1–Atg23 binding, which is required for the recruitment of Atg9 vesicles to the PAS (Yao et al. 2023). TOR also inhibits autophagy in mammals by repressing the activity of death-associated protein 1 (DAP1), which negatively regulates autophagic flux (Koren et al. 2010). However, as no homologs of DAP1 (GenBank: NP_004385.1) have been identified in plants, whether TOR negatively regulates plant autophagy via multiple mechanisms also requires further investigation. In animals, mTORC1 also negatively regulates autophagy at the transcriptional level by phosphorylating Transcription Factor EB, the master transcription factor of autophagy and lysosome genes, at multiple sites (Ciazynska 2023; Martina et al. 2012). This phosphorylation promotes its nuclear export and shuts down the transcription of autophagy genes. By contrast, how TOR regulates autophagy in plants at the transcriptional level is still unclear.

## SnRK1 positively regulates autophagy in plants

**Fig. 2:**
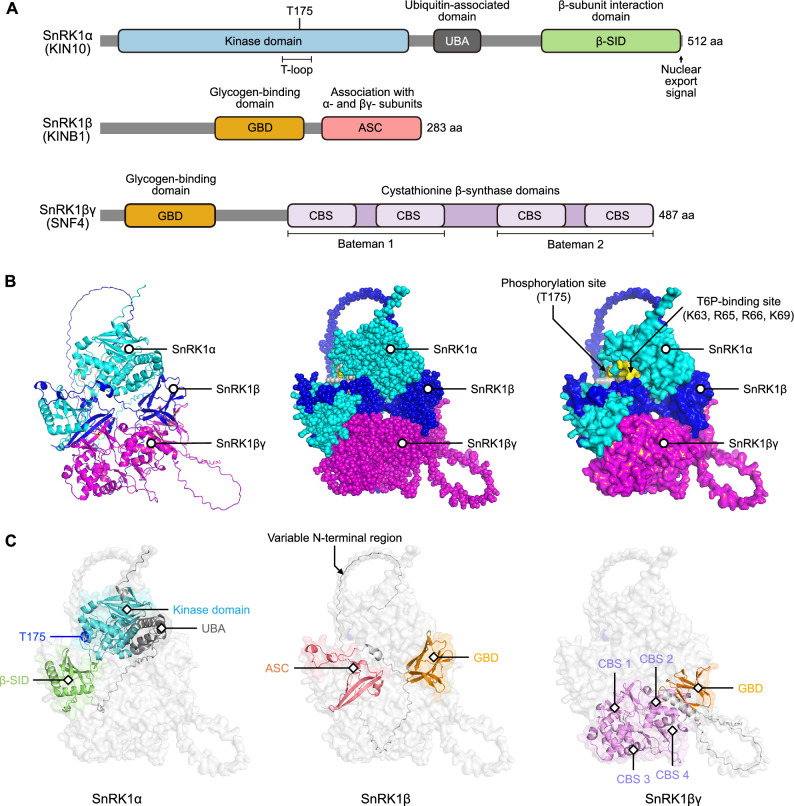
The SnRK1 heterotrimeric complex in plants. **A** Structural features of the SnRK1 subunits in *Arabidopsis*: SnRK1α (KIN10, UniProt accession number: Q38997), SnRK1β (KINB1, UniProt accession number: Q84VQ1), and SnRK1βγ (SNF4, UniProt accession number: Q944A6). Functional domains were annotated using InterPro (https://www.ebi.ac.uk/interpro/) and manually verified based on the previous studies (Ghillebert et al. 2011; Zacharaki et al. 2022). The gray blocks represent the overall protein sequences, while colored rectangles or ellipses indicate functional domains. **B** Structural models of the SnRK1 complex in *Arabidopsis*, shown as a cartoon representation (left), a sphere model (middle), and a surface model (right). The models were predicted using AlphaFold3 (https://alphafoldserver.com/) and manually annotated based on the previous studies (Blanford et al. 2024; Crepin and Rolland 2019). The T-loop containing the conserved threonine residue (T175) and the T6P-binding sites (K63, R65, R66, and K69) of SnRK1α are highlighted in the surface model. **C** Functional domain annotations for each SnRK1 subunit based on information from panels **A** and **B**. The light gray background represents the complete SnRK1 complex

SnRK1 is a heterotrimeric kinase complex that serves as a critical energy sensor in plants. This complex is highly conserved in eukaryotes, where it is referred to as Sucrose Non-Fermenting 1 (SNF1) in yeast and AMPK in animals (Hardie 2007). In plants, the SnRK1 complex consists of three subunits: SnRK1α, SnRK1β, and SnRK1βγ (Emanuelle et al. 2015). Notably, in *Arabidopsis*, the SnRK1α subunit is encoded by three paralogs: *KIN10*, *KIN11*, and *KIN12* (also known as *SnRK1α1*, *SnRK1α2*, and *SnRK1α3*). *KIN10* is considered to be the dominant isoform due to its high abundance and widespread expression, whereas *KIN11* exhibits tissue- and condition-specific expression. *KIN12* is generally regarded as a pseudogene (Sun et al. 2024; Wang et al. 2021). As summarized in Fig. 2, the SnRK1α subunit, illustrated as the prototypical KIN10 in Arabidopsis, contains an N-terminal kinase domain, a UBA domain, and a C-terminal β-subunit interaction domain. The N-terminal domain contains a conserved phosphorylation site with a T-loop structure, which acts as a switch between the active and inactive states of the kinase (Ghillebert et al. 2011). The SnRK1β subunit contains a variable N-terminal region, a glycogen-binding domain (GBD), and a regulatory α-CTD (Ghillebert et al. 2011). The β-subunit acts as the scaffold that holds the other two subunits together (Crozet et al. 2014). The SnRK1βγ subunit contains a GBD in the KINβ-like portion of the subunit, while the KINγ-like portion of the subunit contains four cystathionine β-synthase (CBS) domains that serve as potential nucleotide binding sites (Ghillebert et al. 2011). The SnRK1βγ subunit is plant-specific and differs from the single γ-subunit found in animals and yeast (Gutierrez-Beltran and Crespo 2022; Jamsheer et al. 2021). In mammals, the CBS domains in the γ-subunit bind to adenine nucleotides (ATP, ADP, or AMP), which influences the structure of the AMPK complex in response to changes in the relative amounts of ATP, ADP, and AMP, thereby affecting its activity (Jamsheer et al. 2021; Oakhill et al. 2011). Under energy stress, AMP stabilizes AMPK in its active conformation by protecting the kinase activation loop from dephosphorylation by protein phosphatases, thereby maintaining it in its active, phosphorylated state. Conversely, under low AMP/ATP ratios, ATP binding enhances the conformational dynamics of the activation loop, making it more accessible to phosphatases and promoting dephosphorylation and inactivation (Yan et al. 2021).

In contrast to mammalian AMPK, the critical residues required for binding adenine nucleotides are not conserved in plant SnRK1βγ subunits, which likely explains the insensitivity of SnRK1 to AMP and ADP (Emanuelle et al. 2015). Instead, plant SnRK1βγ subunits may rely on alternative cofactors or mechanisms for binding to AMP and ATP, reflecting a unique regulatory adaptation in plants (Emanuelle et al. 2015). Under normal, high-sugar conditions, SnRK1 is maintained in an inactive state, primarily due to the presence of trehalose 6-phosphate (T6P), which acts as a proxy for high cellular sugar levels. T6P binds to the catalytic SnRK1α subunit, preventing the necessary reorientation of its activation loop (T-loop) for activation (Blanford et al. 2024). This binding also lowers the affinity of SnRK1 toward GEMINIVIRUS REP INTERACTING KINASE (GRIK)-type proteins, the kinases responsible for its activation, also known as SnRK1-activating kinases. We recently reported that FLZs repress SnRK1 signaling by inhibiting the phosphorylation of the T-loop in SnRK1α1 under normal conditions (Yang et al. 2023b). As a result, SnRK1 stays inactive, supporting anabolism. During carbon limitation (i.e., the lack of available carbon sources in extended darkness) or energy stress, T6P levels drop, alleviating the inhibition of SnRK1. This relief from inhibition allows GRIKs to phosphorylate the T-loop of SnRK1α at threonine 175 (T175), inducing a conformational change that activates the kinase (Shen et al. 2009). Thus, the SnRK1 complex senses cellular energy status and positively regulates downstream catabolic programs, such as autophagy (González et al. 2020).

**Fig. 3:**
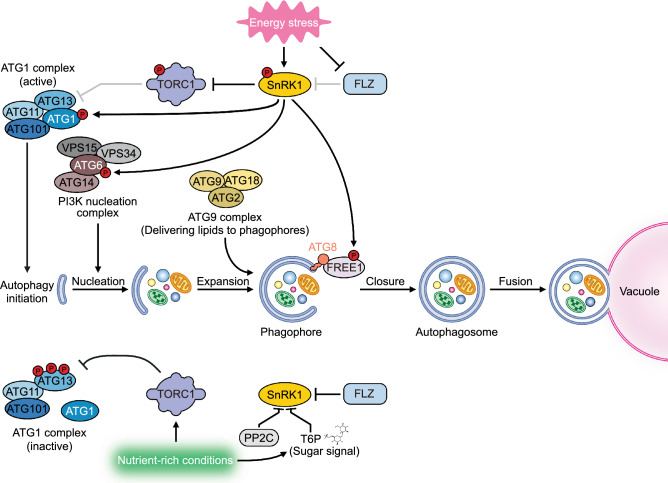
SnRK1 and TORC1 antagonistically regulate autophagy in plants. Under nutrient-replete conditions, TORC1 directly phosphorylates ATG13, preventing its binding to ATG1 (known as ULK1 in animals), thereby inhibiting formation of the ATG1–ATG13 complex and blocking its interaction with SnRK1. SnRK1 activity is also repressed by FLZ, PP2C, and T6P under these conditions. During starvation, SnRK1 becomes activated and phosphorylates RAPTOR to inactivate the TORC1 kinase complex. SnRK1 phosphorylates ATG1, promoting its dissociation from TORC1 and facilitating its association with the site of isolation membrane formation. SnRK1 also directly regulates the phosphorylation of ATG6 in the PI3K complex, which is involved in autophagosome formation. SnRK1 also phosphorylates FREE1, thereby positively regulating autophagosome closure. Arrows represent activation, while T bars represent inhibitory effects. Light gray T bars represent regulatory steps inactive under specific conditions

Autophagy serves as a recycling system to degrade misfolded proteins or damaged organelles to provide resources for intracellular homeostasis under stress or energy-deficient conditions, such as carbon starvation (Fig. 3). AMPK functions as an energy sensor to activate autophagy in both mammalian cells and yeast (Liang et al. 2007; Meley et al. 2006). Overexpressing *KIN10* in *Arabidopsis* enhanced starvation tolerance and extended the plant lifespan, while the *kin10 kin11* double mutant exhibited severe growth defects, including delayed development, premature senescence, and impaired starch mobilization compared to wild-type plants (Baena-González et al. 2007). Arabidopsis plants overexpressing *KIN10* are also hypersensitive to glucose, confirming the central role of SnRK1 in regulating sugar metabolism (Jossier et al. 2009). The sugar signal T6P is thought to inhibit SnRK1, because it coordinates energy availability with plant growth in a manner opposite that of SnRK1 (Blanford et al. 2024; Delatte et al. 2011; Gazzarrini and Tsai 2014; Nunes et al. 2013; Zhang et al. 2009). Type 2C protein phosphatases (PP2Cs) dephosphorylate SnRK1, thereby repressing its kinase function under normal conditions (Rodrigues et al. 2013).

The above findings indicate that SnRK1 is inactive under normal conditions. How does it function as a positive regulator of autophagy in response to carbon starvation? Chen et al. (2017) suggested that KIN10 might positively regulate autophagy by affecting the phosphorylation of ATG1, the initiation complex of the autophagy machinery. Overexpressing *KIN10* in *Arabidopsis* delayed leaf senescence and raised plant tolerance to nutrient limitation (Chen et al. 2017). The stimulation of autophagy during short-term and prolonged carbon starvation is regulated by different components of the autophagy machinery (Huang et al. 2019). SnRK1 may indirectly affect ATG1 phosphorylation under short-term carbon starvation but directly regulate the phosphorylation of ATG6 in the phosphatidylinositol 3-kinase (PI3K) complex to activate autophagy (Huang et al. 2019). A group of mitochondrion-localized FLZ proteins was recently shown to interact with SnRK1 and repress its phosphorylation (Yang et al. 2023b). Carbon starvation inhibits the transcription of *FLZ* genes and promotes FLZ degradation, thereby releasing SnRK1 and positively regulating autophagy (Yang et al. 2023b, 2023c). In addition, SnRK1 can phosphorylate FYVE DOMAIN PROTEIN REQUIRED FOR ENDOSOMAL SORTING 1 (FREE1, also reported as FYVE1) and recruit it to autophagosomes to promote autophagosome closure (Zeng et al. 2023).

## Feedback regulation of autophagy by FLZ proteins in plants

FLZs are a unique class of plant-specific proteins defined by their FCS-like zinc finger domain that mediate protein–protein interactions crucial for their multifaceted roles in plant biology (Chen et al. 2021; Jamsheer et al. 2015; Wang et al. 2023a). These proteins are integral to the complex regulatory networks governing plant growth, development, and stress responses (Jamsheer and Laxmi 2015). Recent studies have highlighted the involvement of FLZs in modulating plant flowering and phytohormone signaling, particularly in response to abscisic acid, a phytohormone required for plant acclimation to environmental stress factors, such as drought and salinity (Jamsheer et al. 2018a; Li et al. 2024a; Yang et al. 2023a).

FLZs regulate energy homeostasis and stress signaling pathways (Jamsheer and Laxmi 2014; Nietzsche et al. 2016, 2014; Yang et al. 2023b). FLZs can interact with SnRK1.1 and SnRK1.2 through their FCS-like zinc finger domains (Jamsheer et al. 2018b). FLZs bind to the UBA domain of SnRK1.1, which is crucial for maintaining T-loop phosphorylation, thereby influencing SnRK1.1 activation (Yang et al. 2023b). Previous studies have demonstrated that FLZs suppress SnRK1 signaling. For example, Arabidopsis *flz6.1* and *flz10.1* mutants exhibit significantly increased SnRK1.1 phosphorylation at T175, while *FLZ*-overexpressing lines, such as *AtFLZ13-OE* and *AtFLZ14-OE*, show markedly reduced phosphorylation (Jamsheer et al. 2018a; Yang et al. 2023b). Additionally, *FLZ* overexpression suppresses the transcriptional activation of the downstream target gene *DIN6* by SnRK1 (Yang et al. 2023b). FLZ3 also inhibits the phosphorylation of the upstream kinase GRIK2, thereby affecting SnRK1 activation (Bortlik et al. 2023).

A recent study revealed that 7 of the 19 FLZ proteins in *Arabidopsis* contain an ATG8-interacting motif (AIM), through which they can interact with ATG8. Under energy-sufficient conditions, FLZs inhibit SnRK1 activity. However, during energy limitation, FLZs are rapidly degraded via the ATG8-mediated autophagy pathway, alleviating their inhibition of SnRK1. This activation of SnRK1 further enhances autophagy, creating a positive feedback loop that fine-tunes energy homeostasis (Yang et al. 2023c).

From an evolutionary perspective, FLZs likely originated in the bryophyte *Marchantia polymorpha*. MpFLZ1 interacts with MpSnRK1.1, indicating that the FLZ–SnRK1 regulatory module is conserved in land plants. However, MpFLZ1 does not interact with MpATG8, and fern FLZs lack the AIM. The emergence of AIM-containing FLZ proteins in gymnosperms suggests that the ATG8–FLZ–SnRK1 regulatory axis might be a conserved feature in seed plants that evolved as an adaptation to terrestrial environments. This notion is supported by the functional conservation of the ATG8-interacting protein ZmFLZ14 in maize (*Zea mays*), as the *Zmflz14* mutant showed enhanced tolerance to energy deprivation, while *ZmFLZ14* overexpression diminished this tolerance (Yang et al. 2023b). This autophagy-mediated feedback regulation underscores the critical roles of autophagy in plant stress responses and developmental plasticity.

## Key roles of organelle-phagy in plant responses to energy stress

### Mitophagy

**Fig. 4:**
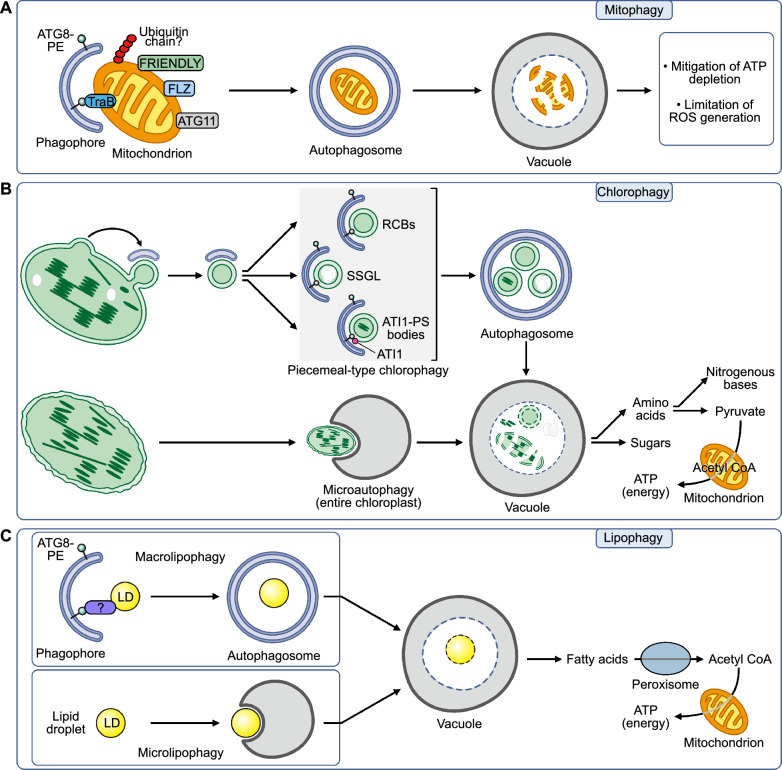
Selective autophagic degradation and energy production in plants under energy stress. **A–C** Diagrams of the autophagic pathways that degrade mitochondria (**A**), chloroplasts (**B**), and lipid droplets (**C**). During mitophagy, TraB family proteins recruit ATG8 to drive autophagosome biogenesis. FRIENDLY, FLZs, and ATG11 also play important roles in mitophagy, but they may not directly act as mitophagy receptors (Duckney et al. 2025). Two forms of chlorophagy have been described: piecemeal-type chlorophagy, which selectively recognizes Rubisco-containing bodies (RCBs), ATG8-INTERACTING PROTEIN 1-plastid-associated (ATI1-PS) bodies, or small starch granule-like (SSGL) bodies; and macrochlorophagy, in which photo-damaged organelles are directly enveloped and transported to the vacuolar lumen (Izumi et al. 2019; Wan and Ling 2022). Like chlorophagy, lipophagy in plants may involve macrolipophagy and microlipophagy, though specific mechanisms remain under investigation (Goodman 2021; He 2022). Question marks indicate unresolved roles and mechanisms of the listed proteins in organelle-phagy

Mitochondria play crucial roles in organismal energy metabolism, survival, and signal transduction. Under carbon starvation conditions, mitochondrial proteins can be transported to the vacuole for degradation in *Arabidopsis* (Fig. 4A), with about 5–10% of all mitochondrial proteins being removed daily via autophagic pathways (Vincow et al. 2019). In animals, the PTEN-induced putative protein kinase 1 (PINK1)/Parkin pathway is responsible for the ubiquitination of outer membrane proteins of depolarized mitochondria (Ashrafi and Schwarz 2013). Subsequently, the ubiquitinated mitochondrial outer membrane proteins are targeted for autophagic degradation by autophagic receptors, such as p62, Optineurin, and Nuclear Dot Protein 52 (Ke et al. 2022). Furthermore, several outer membrane proteins (BCL2-interacting protein 3, NIX, FUN14 domain-containing 1, and Atg32 in yeast) and inner membrane proteins (Prohibitin 2 [PHB2]) can directly interact with ATG8 to facilitate mitochondrial degradation (Wei et al. 2015, 2017).

Plants lack direct homologs of the PINK1/Parkin pathway and known mitophagy receptors of animals, necessitating unique mechanisms for mitochondrial degradation. Therefore, research on mitophagy in plants represents pioneering work rather than a direct replication of studies conducted in animals. Senescence, carbon starvation, and oxidative stress have emerged as the primary triggers for mitophagy. In *Arabidopsis*, the interaction between ATG11 and ATG8 mediates senescence-induced mitophagy, whereas in yeast, Atg11 mediates mitochondrial degradation by interacting with Atg32 (Kanki et al. 2009; Li et al. 2014). FLZs localize to mitochondria and are transported to the vacuole for degradation under carbon starvation; however, whether FLZs participate in mitophagy itself remains unknown (Jamsheer et al. 2018b; Yang et al. 2023b). Membrane contact sites may also be involved in the degradation of depolarized mitochondria. The mitochondrial, outer membrane-localized, TraB family proteins TRB1 and TRB2 interact with the endoplasmic reticulum (ER)-localized proteins VESICLE-ASSOCIATED PROTEIN 27 and ATG8, acting as mitochondrial receptors in plants (Li et al. 2022a). FRIENDLY (FMT) may also be recruited to depolarized mitochondria, preventing abnormal mitochondrial aggregation (Ma et al. 2021). However, the roles of TRB1, TRB2, and FMT in carbon starvation-induced mitophagy require further investigation.

Despite the identification of multiple autophagic receptors and/or adapters that mediate mitophagy in mammals, our knowledge of mitophagy receptors/adapters in plants remains insufficient. Which signals trigger mitophagy during carbon starvation? Mitophagy is primarily associated with energy depletion. SnRK1 inhibits cellular growth while promoting stress responses. SnRK1 regulates overall autophagic levels through the phosphorylation of ATG1 and ATG6, and it is likely that SnRK1 plays a significant role in mitophagy induced by starvation.

### Chlorophagy

The protein content of chloroplasts accounts for about 70–80% of all proteins in leaves, serving as a crucial nitrogen storage pool (Izumi and Ishida 2019). Chlorophagy, the degradation of chloroplasts, is primarily induced by carbon starvation or photodamage (e.g., ultraviolet-B and high light) (Wang et al. 2023b). During carbon starvation, chloroplast proteins are degraded, and chloroplast components are transported to the vacuole for degradation (Fig. 4B). This response to carbon starvation is blocked in autophagy mutants, indicating that these components are degraded via the autophagy pathway (Izumi et al. 2017; Wijerathna-Yapa et al. 2021). Immuno-electron microscopy observations of senescing leaves from wheat (*Triticum aestivum*) plants revealed that the normally chloroplast stroma-localized protein Ribulose-1,5-bisphosphate carboxylase/oxygenase (Rubisco) accumulates in Rubisco-containing bodies (RCBs); these vesicles, ranging from 0.4 to 1.2 μm in diameter, are abundant in the cytoplasm and vacuole. This observation suggested that RCBs may participate in chloroplast degradation (Ishida et al. 2008). Darkness promotes the aggregation of RCBs in vacuoles, which is not observed in the Arabidopsis *atg5-1* mutant (Ishida et al. 2008). In this mutant, CT-GFP, comprising the chloroplast transit peptide from Arabidopsis RECA fused to green fluorescent protein (GFP) for targeting to the chloroplast stroma, abnormally localizes to the periphery of chloroplasts, forming a tubular structure after dark induction. Thus, autophagy does not appear to be involved in RCB formation but is essential for the relocation of RCBs from chloroplasts to the vacuole (Ishida et al. 2008).

The chloroplast outer membrane components TRANSLOCON AT THE OUTER ENVELOPE MEMBRANE OF CHLOROPLASTS 75 (Toc75) and Toc33 accumulate in the autophagy-deficient mutant *atg7-2*, underscoring the role of autophagy in regulating the degradation of chloroplast outer membrane components (Wan et al. 2023). In addition, loss of function of the Endosomal Sorting Complex Required for Transport (ESCRT)-III component CHARGED MULTIVESICULAR BODY PROTEIN 1 in *Arabidopsis* led to the formation of tubular structures, with chloroplast proteins visibly accumulating within them (Spitzer et al. 2015). The formation of these punctate structures on the chloroplast outer membrane does not depend on autophagy, but their relocation from the chloroplast for degradation does rely on autophagy (Spitzer et al. 2015). Carbon starvation stress plays a significant role in inducing RCB formation, and sugar supplementation can alleviate dark-induced chlorophagy. Moreover, electron microscopy revealed a starch granule-like structure in the cytoplasm, enveloped by autophagosomes. This structure, marked by a fusion between granule-bound starch synthase and the yellow fluorescent protein (GBSS1-YFP), forms small bodies outside chloroplasts under dark conditions and colocalizes with ATG8 in *Arabidopsis* (Wang et al. 2013).

ATG8-INTERACTING PROTEIN1 (ATI1) and its paralog ATI2, the first identified chloroplast-selective autophagy receptors, localize to the ER and chloroplast outer membranes via their C-terminal transmembrane domains, with N-terminal AIMs exposed to the cytoplasm. Under carbon starvation, ATI1, and likely ATI2, accumulate on the chloroplast outer membrane, forming 1-μm plastid-associated bodies (ATI-PS bodies) and ER-associated puncta (ATI-ER bodies) (Michaeli et al. 2014). ATI-PS bodies, labeled by ATI1-GFP, partially colocalize with the stromal marker CT-GFP on plastids and in the vacuole, suggesting a role in delivering stromal proteins similar to RCBs, though their release from chloroplasts is autophagy independent (Michaeli et al. 2014). However, the release of ATI-PS bodies from chloroplasts appears to be independent of the autophagic mechanism. Additionally, NEXT TO BRCA1 (NBR1) couples K63-ubiquitinated TOC proteins to ATG8, mediating the selective autophagic degradation of chloroplasts (Wan et al. 2023). In light of these results, chlorophagy is a sophisticated, autophagy-mediated process critical for plant adaptation to stress, enabling nutrient recycling through specialized structures like RCBs and receptors such as ATI1 and ATI2. While some steps, like RCB formation and ATI-PS body release, occur independently of autophagy, the process as a whole relies on autophagy for efficient degradation. Recent innovations, including synthetic receptors, underscore chlorophagy’s potential for agricultural applications (Liu et al. 2025). Future research exploring the signals initiating chlorophagy and its selective targeting of chloroplast components could unlock new strategies for developing stress-resilient crops.

### Lipophagy

Together with sugars and proteins, lipids serve as essential carbon reserves in plants. As carbon storage molecules, lipids have a high potential to generate energy in the form of ATP (Fig. 4C). When the carbohydrate supply is impaired—such as during carbon starvation, senescence, drought, or hypoxia—lipids can become alternative respiratory substrates for energy production, both during development and in response to environmental stress (Barros et al. 2020).

Lipids constitute about 60% of the total weight of seeds and are primarily stored as lipid droplets (LDs). Triacylglycerols are the main components of LDs, followed by phospholipids (Bouchnak et al. 2023). During seed germination or carbon starvation, these lipids are mobilized to provide energy to the plant. Two primary mechanisms mediate lipid mobilization: lipid hydrolysis via lipases on the surface of LDs and LD transport to vacuoles for degradation, a program known as lipophagy (Yoshitake et al. 2019). Disrupting lipid degradation can significantly impair seed germination, and autophagy mutants exhibit an imbalance in lipid metabolism in leaves (Barros et al. 2021).

During seed germination, LDs can be observed within vacuoles. Furthermore, LDs labeled with OLE1–GFP, a fusion between OLEOSIN 1 and GFP, colocalized with the autophagosome marker ATG8e after carbon starvation, providing evidence for the involvement of autophagy in the degradation of LDs (Fan et al. 2019). Similarly, maize *atg12* mutants, deficient in autophagy, accumulate significantly higher levels of lipid breakdown products in leaves under fixed-carbon starvation, indicating impaired autophagic turnover of LDs compared to wild-type plants (McLoughlin et al. 2020). While it is well established that LDs are degraded through autophagic pathways during seed germination and carbon starvation, the exact molecular mechanisms remain to be fully explored. Components of the ESCRT machinery, such as FREE1, may play essential roles in the autophagic degradation of plant LDs. The degradation of LDs is impaired in the *free1* mutant, suggesting a key regulatory role for FREE1 (Huang et al. 2022). FREE1 was recently shown to interact with ATG8, which functions in autophagosome fusion, further implicating FREE1 in the autophagic degradation of LDs (Zeng et al. 2023). However, the precise mechanism by which FREE1 mediates LD degradation remains an area for further investigation.

### Selective autophagy of other organelles

In addition to mitophagy, chlorophagy, and lipophagy, selective autophagy also directs the degradation of other organelles, such as the ER, the Golgi apparatus, and peroxisomes (Otegui et al. 2024). Their degradation is also essential for cellular homeostasis, especially under stress conditions, by ensuring the removal of damaged or superfluous organelles. Here, we summarize the current understanding of selective autophagy pathways targeting the ER, Golgi, and peroxisomes in plants.

The ER is a dynamic organelle responsible for protein synthesis, lipid metabolism, and calcium storage. Under stress conditions, such as nutrient limitation or the accumulation of misfolded proteins, the ER undergoes selective autophagy, which is known as ER-phagy, to maintain its functionality and prevent cellular damage. ER-phagy involves the recognition of damaged or excess ER compartments, their fragmentation, and their subsequent engulfment by autophagosomes for degradation in the vacuole. In plants, ER-phagy is triggered by stress factors, including carbon and phosphate starvation, as well as ER stress induced by protein-folding inhibitors such as tunicamycin (Zeng et al. 2019). The initiation of ER-phagy is mediated by specific ER-phagy receptors that interact with ATG8, facilitating the selective degradation of ER components. One well-characterized ER-phagy receptor in plants is ATI1, which localizes to both the ER and chloroplasts. ATI1 forms ATI-PS bodies under starvation conditions that are involved in the degradation of ER and chloroplast components (Michaeli et al. 2014; Wu et al. 2021). Another ER-phagy receptor, ATI3, distinct from ATI1 and its paralog ATI2, is critical for ER stress tolerance and interacts with UBP1-ASSOCIATED PROTEIN 2C, an ER-associated degradation (ERAD) regulator, linking ER-phagy to ERAD (Zhou et al. 2018). Additionally, RETICULON 1 (RTN1) and RTN2 in maize modulate ER-phagy, particularly in the endosperm, where the ER undergoes extensive remodeling during seed development (Zhang et al. 2020). ER-phagy is also connected to the ER-folding machinery via Secretory 62 (Sec62), a component of the translocon complex. In *Arabidopsis*, Sec62 homologs contribute to ER homeostasis by mediating the degradation of excess ER generated during stress recovery (Hu et al. 2020). Furthermore, the cytosolic protein C53 was identified as an ER-phagy receptor that senses ribosome stalling and triggers the degradation of incomplete polypeptides via autophagy (Stephani et al. 2020). These findings highlight the intricate regulation of ER-phagy and its role in maintaining ER integrity under stress conditions.

The Golgi apparatus is a central hub for protein sorting, modification, and secretion. Like other organelles, the Golgi is susceptible to damage under stress conditions, and its selective degradation, known as Golgiphagy, is essential for maintaining cellular function. Golgiphagy involves the encapsulation of Golgi fragments within autophagosomes, which are then delivered to the vacuole for degradation. In plants, Golgiphagy is less well understood compared to other forms of selective autophagy. However, recent studies have provided insights into the mechanisms underlying Golgi degradation. Under acute heat stress (45 °C for 5 min), the Golgi undergoes structural disruption, with cisternae swelling and vacuolating to form hypertrophied stacks, likely impairing its protein sorting and secretion functions (Zhou et al. 2023). ATG8 is recruited to these swollen Golgi membranes, where its lipidation anchors it to facilitate the formation of single-membrane vesicles, distinct from canonical autophagosomes, in a process involving clathrin components that aids Golgi reassembly. The Arabidopsis *atg5* mutant, defective in ATG8 lipidation and its translocation to the Golgi, exhibits delayed Golgi recovery, failing to restore normal stacked cisternal structure and function after heat stress, highlighting the importance of this ATG8-mediated process for Golgi resilience (Zhou et al. 2023). Nutrient limitation, particularly sucrose starvation, has also been shown to induce Golgiphagy in plants. In tobacco (*Nicotiana tabacum*) BY-2 cells, sucrose starvation triggered the degradation of *trans*-Golgi network proteins, such as SUCROSE TRANSPORTER 2 and SYNTAXIN OF PLANTS 41, in an autophagy-dependent manner (Oda et al. 2020). Despite these advances, the identification of specific Golgiphagy receptors and the underlying molecular mechanisms remain areas of active research.

Peroxisomes are dynamic organelles involved in various metabolic pathways, including photorespiration and fatty acid oxidation. Due to their role in ROS metabolism, peroxisomes are prone to oxidative damage, requiring their selective degradation via autophagy, known as pexophagy. Pexophagy can occur through two major pathways: macropexophagy, where peroxisomes are sequestered by autophagosomes; and micropexophagy, where peroxisomes are directly engulfed by the vacuolar membrane. In plants, pexophagy is essential for the turnover of damaged peroxisomes and the transition between different peroxisomal functions, such as the conversion of glyoxysomes to leaf peroxisomes during seedling greening. ATG2, ATG7, and ATG18a are essential for pexophagy in *Arabidopsis*, as single mutants lacking any one of these genes exhibit peroxisome aggregation and accumulate excess peroxisomes due to impaired degradation of damaged peroxisomes (Shibata et al. 2013). The selective autophagy receptor NBR1 has been implicated in pexophagy under oxidative stress conditions, such as cadmium stress, where it associates with peroxisomes and interacts with ATG8 (Calero-Muñoz et al. 2019). However, NBR1-independent pexophagy pathways also exist, suggesting multiple mechanisms for peroxisome degradation. LON PROTEASE 2 (LON2), a peroxisomal matrix protein with chaperone and peptidase activities, is a regulator of pexophagy. LON2 deficiency leads to excessive pexophagy, indicating that LON2 suppresses peroxisome degradation (Goto-Yamada et al. 2014). Additionally, the actin-related protein (ARP2/3) complex localizes to peroxisomes and interacts with ATG8, suggesting a role for the cytoskeleton in pexophagy (Martinek et al. 2023). These findings highlight the complexity of pexophagy and its importance in peroxisome homeostasis under stress conditions.

## Concluding remarks

Studies investigating the role of autophagy in regulating plant cell energy balance have highlighted how this catabolic pathway is a crucial mechanism for energy homeostasis. When plants experience low-energy supply, they initiate autophagy to selectively degrade cellular components, providing the necessary energy to sustain life. Conversely, under conditions of energy abundance, plants must inhibit autophagy to prevent interference with normal physiological functions. While our understanding of autophagy triggers in plants has advanced, research on the upstream mechanisms that initiate it and the cargo recognition receptors involved is still in its early phases.

Mitochondria and chloroplasts are central to the energy metabolism of plant cells, but the mechanisms governing the exchange of energy metabolites between these organelles under stress conditions remain unclear. Autophagy plays a vital role in supporting the homeostasis of both mitochondria and chloroplasts. However, to date, no universally accepted autophagy receptors specific to these organelles have been identified in plants. Conflicting findings on receptors like NBR1, which may partially mediate chlorophagy but not mitophagy, highlight ongoing debates in the field (Jung et al. 2019; Lee et al. 2023; Wan et al. 2023). For example, NBR1 was proposed as a receptor for chlorophagy capable of partially encapsulating damaged chloroplasts (Wan et al. 2023). However, studies on the *nbr1* mutant suggest that ATG8 is still able to envelop chloroplasts (Lee et al. 2023), pointing to a more complex mechanism. Furthermore, evidence suggests that NBR1 does not participate in mitophagy (Jung et al. 2019). These discrepancies highlight the challenges ahead in identifying definitive autophagy receptors for mitochondria and chloroplasts, making this an ongoing and critical area of research.

To deepen our understanding of the relationship between energy metabolism and autophagy, several key research directions warrant further investigation. TOR and SnRK1 are both central regulators of autophagy, but their interplay remains poorly understood. Elucidating how these pathways coordinately regulate autophagy in response to energy stress could reveal previously unknown mechanisms by which plants fine-tune homeostasis under energy stress. While autophagy is known to degrade a wide range of cellular components, it is unclear whether certain substrates are preferentially targeted under energy stress. Identifying these substrates could provide valuable insight into how cells prioritize energy sources during stress. Additionally, investigating whether plants have unique mechanisms for regulating the TOR–SnRK1 autophagy axis, such as through light signaling or chloroplast energy status (e.g., ATP/NADPH ratios), is a priority. Finally, investigating the potential to improve crop resilience and productivity by modulating the TOR–SnRK1 autophagy axis and FLZs represents a promising avenue for future research (Gross et al. 2025; Guan et al. 2025; Petersen et al. 2024; Thanthrige et al. 2021).

## Data Availability

Data sharing is not applicable to this article as no datasets were generated or analyzed during the current study.
